# Relationship between Driving Pressure and Mortality in Ventilated Patients with Heart Failure: A Cohort Study

**DOI:** 10.1155/2021/5574963

**Published:** 2021-11-29

**Authors:** Qilin Yang, Jiezhao Zheng, Xiaohua Chen, Weiyan Chen, Deliang Wen, Xuming Xiong, Zhenhui Zhang

**Affiliations:** Department of Critical Care, The Second Affiliated Hospital of Guangzhou Medical University, No. 250 Changgang East Road, Haizhu District, Guangzhou, China

## Abstract

**Background:**

Heart failure (HF) is a leading cause of mortality and morbidity worldwide, with an increasing incidence. Invasive ventilation is considered to be essential for patients with HF. Previous studies have shown that driving pressure is associated with mortality in acute respiratory distress syndrome (ARDS). However, the relationship between driving pressure and mortality has not yet been examined in ventilated patients with HF. We assessed the association of driving pressure and mortality in patients with HF.

**Methods:**

We conducted a retrospective cohort study of invasive ventilated adult patients with HF from the Medical Information Mart for Intensive Care-III database. We used multivariable logistic regression models, a generalized additive model, and a two-piecewise linear regression model to show the effect of the average driving pressure within 24 h of intensive care unit admission on in-hospital mortality.

**Results:**

Six hundred and thirty-two invasive ventilated patients with HF were enrolled. Driving pressure was independently associated with in-hospital mortality (odds ratio [OR], 1.12; 95% confidence interval [CI], 1.06–1.18; *P* < 0.001) after adjusted potential confounders. A nonlinear relationship was found between driving pressure and in-hospital mortality, which had a threshold around 14.27 cmH_2_O. The effect sizes and CIs below and above the threshold were 0.89 (0.75 to 1.05) and 1.17 (1.07 to 1.30), respectively.

**Conclusions:**

There was a nonlinear relationship between driving pressure and mortality in patients with HF who were ventilated for more than 48 h, and this relationship was associated with increased in-hospital mortality when the driving pressure was more than 14.27 cmH_2_O.

## 1. Introduction 

Heart failure (HF) is a syndrome caused by structural or functional cardiac abnormalities that lead to elevated intracardiac pressures or a reduced cardiac output. HF is a leading cause of mortality and morbidity worldwide [1–3]. Previous studies have shown that the prevalence of HF increased by as much as 30% in Medicare beneficiaries from 1994 to 2003 [4]. In the study of Atherosclerosis Risk in Communities (ARIC) of the National Heart, Lung, and Blood Institute (NHLBI), the 30-day, 1-year, and 5-year case fatality rates after hospitalization for HF were up to 10.4%, 22%, and 42.3%, respectively [5]. HF is commonly complicated by pulmonary edema. Approximately half of patients with acute respiratory failure with cardiogenic shock and acute myocardial infarction require ventilation, especially invasive mechanical ventilation [5–8].

Driving pressure (plateau pressure (Pplat) – positive end-expiratory pressure (PEEP)) has recently received attention and has been widely studied as a static index indicating the inflation characteristics of pulmonary alveoli during the individual respiratory cycle [9, 10]. The lung elastance is computed as the ratio between driving pressure and tidal volume (*Vt*), so the driving pressure can also be calculated as *Vt∗* respiratory system elastance. Therefore, by assuming that the tidal volume is fixed during mechanical ventilation, it is clear to understand how the driving pressure is strictly correlated with the mechanical features of the respiratory system. Moreover, ventilated patients occasionally present with ventilator-induced lung injury (VILI), which increases mortality [11]. Some studies have suggested that VILI is partly caused by respiratory system elastance and have shown that driving pressure is a good predictor of VILI [12, 13].

The relationship between driving pressure and outcomes has gained increasing interest, especially in acute respiratory distress syndrome (ARDS) [14, 15]. Relevant studies have shown that lower driving pressure could decrease lung overstress and was associated with lower mortality in patients with ARDS [14–17]. However, the increased risk of poor prognosis associated with high drive pressure has been observed not only in patients with ARDS but also in ventilated patients, which includes patients with severe pneumonia without ARDS [18] and surgical patients with healthy lungs during the perioperative period [19–21]. Even so, studies on driving pressure and patients with HF are limited. Cross et al. found that, during the graded exercise test, patients with HF had decreased dynamic lung volumes and dynamic lung compliance and increased breath rate, compared to healthy controls [22], suggesting that higher driving pressure is generated during mechanical ventilation. We hypothesized that driving pressure is also associated with outcomes in ventilated patients with HF, although the previous study showed that driving pressure was not associated with in-hospital mortality in non-ARDS patients.

This study mainly assessed the association of driving pressure and mortality in patients with HF and supported the suggestion, using driving pressure limiting strategy in patients with HF during invasive ventilation.

## 2. Materials and Methods

### 2.1. Database

This retrospective observational cohort study used the Medical Information Mart for Intensive Care-III (MIMIC-III version 1.4) database. The MIMIC-III is a real-world and publicly accessible database containing information on more than 60,000 adult intensive care unit (ICU) stays in Beth Israel Deaconess Medical Center between 2001 and 2012 [23]. The Institutional Review Boards (IRBs) at both the Massachusetts Institute of Technology (MIT) and the Beth Israel Deaconess Medical Center (BIDMC) approved the use of the data for research. The study protocol was approved by the Medical Ethics Committee of the Second Affiliated Hospital of Guangzhou Medical University (2019-ks-11).

### 2.2. Study Population

Patients with HF were considered eligible for our study. The diagnosis of HF was based on the International Classification of Disease, Ninth Revision (ICD-9) categorized by Quan et al. [24]. As HF may not always be listed as the principal diagnosis, we also included records with HF in any of the first five diagnostic positions according to the diagnostic sequence. The inclusion criteria were as follows: (1) patients were aged ≥18 years; (2) patients received invasive ventilation for at least 48 h; and (3) only the first ICU admission was considered [25].

Patients who were extubated or extirpated during the first 48 h were excluded. Patients who had incomplete datasets or datasets that did not suffice to calculate driving pressure were also excluded [25].

### 2.3. Exposure Variable

PEEP and plateau pressure were extracted as the time-weighted values during the first 24 h of mechanical ventilation and were calculated by averaging the result of the subtraction of PEEP from plateau pressure at each minute during the first 24 h of ventilation. The minute-by-minute value was obtained by carrying each observation forward until the next observation was recorded [26].

### 2.4. Covariates

The relevant variables for this study were extracted from the previous report. These variables are commonly used in studies of the relationship between driving pressure and outcomes [14, 26–28]. The following variables were included: demographic characteristics, sequential organ failure assessment (SOFA) score, simplified acute physiology score (SAPS), and Elixhauser comorbidity score. N-terminal pro-B-type natriuretic peptide (NT-proBNP) and vital signs (mean arterial pressure (MAP), heart rate, and central venous pressure (CVP)) were recorded 24 h before and after ICU admission. The laboratory test results from the first day of admission were required [white blood cell (WBC) count, hemoglobin, creatinine, pondus hydrogenii (PH), lowest partial pressure of oxygen in arterial blood (PaO_2_), highest partial pressure of carbon dioxide (PaCO_2_) in arterial blood, and lowest PF ratio]. Some settings and observations needed during mechanical ventilation were considered, including ventilation mode and tidal volume. However, NT-proBNP and CVP were not collected for more than half of the included patients. Dummy variables were used to indicate missing NT-proBNP and CVP values [29, 30]. Body mass index (BMI), calculated as the weight (kg) divided by the square of height (m) [31], and predicted body weight follow the standard formula recommended in the ventilation strategy for patients with ARDS.

### 2.5. Outcomes

The primary outcome was in-hospital mortality.

### 2.6. Statistical Analysis

Patient characteristics were analyzed according to the driving pressure tertials. Categorical variables are expressed as numbers and percentages. Continuous variables are expressed as mean and standard deviation (SD) for normal distributions or median and interquartile range (IQR) for skewed distributions. We used the chi-square test, one-way ANOVA, and Kruskal-Wallis test for the comparison of categorical, normally distributed, and nonnormally distributed continuous variables, respectively.

Univariate linear regression analyses and multivariable logistic regression analyses were performed to evaluate the associations between driving pressure and in-hospital mortality. According to the recommendation of the STrengthening the Reporting of OBservational studies in Epidemiology (STROBE) statement [32], analyses were first performed without adjustment. Further analyses cumulatively included adjustment for age, sex (minimally adjusted model), heart rate, MAP, WBC, hemoglobin, creatinine, CVP, NT-proBNP, PEEP, PH, PaO_2_, PaCO_2_, PF ratio, Elixhauser score, SOFA score, and SAPS score (fully adjusted model).

We used a generalized additive model (GAM) to identify the nonlinear relationship [33, 34]. If a nonlinear correlation was observed, a two-piecewise linear regression model was conducted to calculate the threshold effect of the driving pressure on in-hospital mortality in terms of the smoothing plot. When driving pressure and in-hospital mortality were evident in the smoothed curve, the recursive method automatically calculated the threshold, and the maximum model likelihood was used [33]. We performed tests for linear trend by entering the median value of each category of driving pressure as a continuous variable in the models [35].

All analyses were performed using the statistical software package R. version 3.4.3 (R Foundation for Statistical Computing, Vienna, Austria) and Free Statistics software version 1.4. *P* values < 0.05 (two-sided) were considered statistically significant.

### 2.7. Sensitivity Analysis

We excluded patients who were ventilated before ICU admission and patients who received ventilation through tracheostomy cannula at any time during the first 48 h of ventilation as a sensitivity analysis. As a result, patients with chronic obstructive pulmonary disease (COPD) were also excluded because they may have suffered from right HF [25, 26].

## 3. Results

### 3.1. Participants Selection

**Figure 1:**
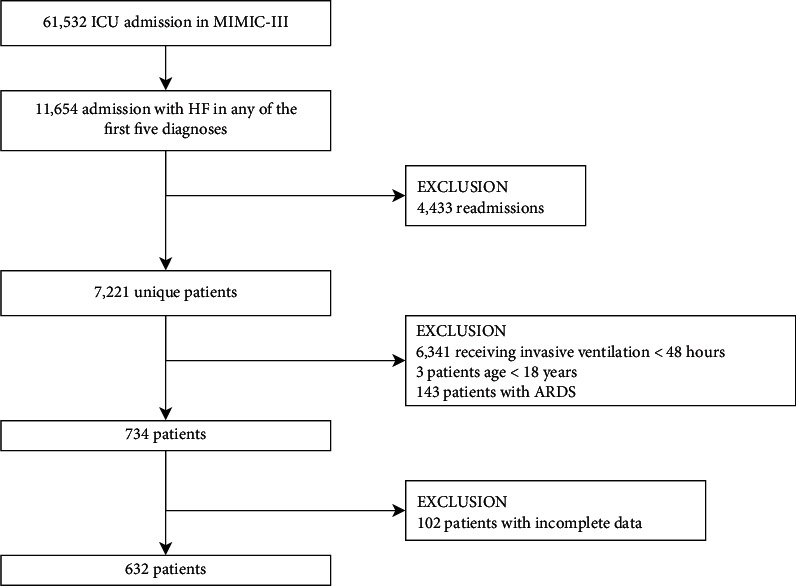
Flowchart of the study cohort.

Of a total of 61,532 MIMIC-III admissions, 7,221 patients with HF were identified.  Figure 1 presents a flowchart of the study. Finally, 632 patients with complete ventilatory data were included.

### 3.2. Baseline Characteristics

The baseline characteristics are presented in Table 1. Patients enrolled were grouped by the tertials of driving pressure as follows: low group, ≥5.00 to ≤12.85 cmH_2_O; middle group, ≥12.86 to ≤16.05 cmH_2_O; and high group, ≥16.10 to ≤35.68 cmH_2_O. The mean age of all participants was 71.4 ± 13.5 years, and 51.11% were male. Some differences existed between the driving pressure groups with respect to various covariates (BMI, SAPS score, Elixhauser score, CVP, PEEP, driving pressure, hemoglobin, and VT/PBW). Furthermore, in terms of ventilation mode, the proportions of patients using controlled mechanical ventilation in each group were similar, over one-third, and there was no significant difference between groups (*P*=0.996).

### 3.3. Outcomes

The overall in-hospital mortality was 28.64%. The in-hospital mortality of patients with low, middle, and high driving pressure was 27.19%, 21.13%, and 38.12%, respectively.

### 3.4. Driving Pressure and In-Hospital Mortality

The results of the univariate analysis of covariates and in-hospital mortality are shown in Supplementary Table 1. Multivariable logistic regression analyses were used to assess the associations between driving pressure and in-hospital mortality (Table 2). In the minimally adjusted model adjusted for age and sex, driving pressure was positively associated with in-hospital mortality (odds ratio [OR], 1.09; 95% confidence interval [CI], 1.04–1.14; *P*=0.0002). Even after adjusting for all potential covariates (Table 2, fully adjusted model), the association remained significant with driving pressure expressed as a continuous variable (OR, 1.12; 95% CI, 1.06–1.18; *P* < 0.0001; Table 2).

When driving pressure entered the fully adjusted model as a categorized variable, the changing trend of the effective value in different driving pressure groups was nonequidistant. The middle and high driving pressure groups had an in-hospital mortality risk that was 26% lower (OR, 0.74; 95% CI, 0.44–1.23; *P*=0.242) and 126% higher (OR, 2.26; 95% CI, 1.36–3.77; *P*=0.002), respectively, compared to low driving pressure patients. *P* for trend = 0.002 (Table 2).

### 3.5. Nonlinear Relationship between Driving Pressure and In-Hospital Mortality

**Figure 2:**
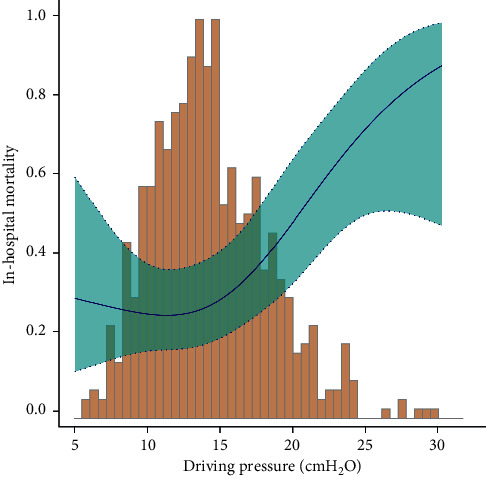
Nonlinear dose-response relationship between driving pressure and in-hospital mortality. Adjustment factors included age, sex, BMI, heart rate, MAP, WBC, hemoglobin, creatinine, CVP, NT-proBNP, PEEP, PH, PaO_2_, PaCO_2_, PF ratio, ventilation mode (CMV), VT/PBW, Elixhauser score, SOFA score, and SAPS score. The blue line and green area represent the estimated values and their corresponding 95% confidence intervals, respectively.

We observed a nonlinear dose-response relationship between driving pressure and in-hospital mortality after adjusting for some covariates (Figure 2). Using a two-piecewise linear regression model, we found that the threshold of driving pressure was 14.27 cmH_2_O (Table 3). Above the threshold, the in-hospital mortality rose rapidly (OR, 1.17; 95% CI, 1.07–1.30; *P* < 0.001; Table 3 and Figure 2), and, below the threshold, the estimated dose-response curve was consistent with a horizontal line (OR = 0.89; 95% CI, 0.75–1.05; *P*=0.176; Table 3 and Figure 2).

### 3.6. Sensitivity Analysis

In the sensitivity analysis, we removed 28 patients who received ventilation through tracheostomy cannula at any time during the first 48 h of ventilation and excluded 171 patients who were ventilated before ICU admission, as well as 47 COPD patients. The association between driving pressure and mortality remained reliable (Supplementary Tables 2–4).

## 4. Discussion

In this retrospective cohort study, driving pressure was independently associated with in-hospital mortality in invasive ventilated patients with HF. We observed a positive association between driving pressure and risk of mortality in patients with HF who underwent ventilation for more than 48 h. The association was reliable and independent of essential covariates and confounders. To the best of our knowledge, this is the first report of an association between driving pressure and mortality in ventilated patients with HF.

Furthermore, the changing trend of the effective value in different driving pressure groups was nonequidistant, which suggested that the association between driving pressure and in-hospital mortality was likely to be nonlinear. Our study used a generalized additive model and found a nonlinear relationship between driving pressure and mortality in patients with HF. Nevertheless, it was significantly different that driving pressure affected in-hospital mortality in patients with HF when it was below or above the threshold of 14.27 cmH_2_O. As assessed at baseline, a positive association was only found for driving pressure above the threshold; otherwise there was no statistical significance, suggesting that a threshold effect was present.

In ICU, low tidal volume ventilation was suggested to avoid ventilator-induced lung injury [36–38]. However, it is important to clarify that ventilator-induced lung injury is not only due to high tidal volume but also due to multiple factors, including airway pressure, PEEP, respiratory rate, and biologic factors [39]. The driving pressure, which is the difference between the plateau pressure and the level of positive end-expiratory pressure, is a good composite indicator. Amato et al. found that driving pressure was the variable that best correlated with survival in patients with ARDS [14]. In Amato's study, each cmH_2_O increment of driving pressure was associated with a hazard ratio (HR) of 1.049 in terms of the risk of death in patients with ARDS [14], which was similar to the result reported by Guérin et al. [27]. In our cohort, with each cmH_2_O increment, driving pressure was associated with an OR of 1.12 in the risk of in-hospital mortality in patients with HF. This may be due to the pulmonary edema which manifests in patients with HF and ARDS despite their different mechanisms [22]. Lung compliance has been found to be significantly reduced when patients suffer from pulmonary edema, while higher driving pressure promotes lung overstress, leading to lung injury [16]. Therefore, it is reasonable that driving pressure may affect the mortality of patients with HF in the same manner as in patients with ARDS. Nevertheless, it is worth noting that the relationship may be biased in obese patients because of the unique mechanics of the respiratory system (lower total lung capacity and functional residual capacity and higher pleural pressure and airway resistance) [40]. The baseline characteristics of the current patients indicated that the BMI was higher in the high driving pressure cohort than in the other two groups, which can be interpreted as the stiffness of the chest-wall rather than the worsening in the lung elastance [41].

This study is the first to report a nonlinear relationship between driving pressure and mortality. We calculated a threshold of approximately 14 cmH_2_O, while the effect below and above the threshold was completely different. This threshold was similar to the cutoff values reported in several previous studies focused on outcomes of patients with ARDS. The cutoff values of driving pressure were 13 cmH_2_O [27], 14 cmH_2_O [42], and 15 cmH_2_O [14], respectively. A recent prospective longitudinal cohort study of patients with ARDS showed that the lung function and lung fibrosis index at 6 months were worse in high driving pressure group (≥13 cmH_2_O) than in the low driving pressure group (<13 cmH_2_O) [43]. Based on the results of our study, we inferred that the threshold could explain why researchers chose driving pressure around 14 cmH_2_O as a cutoff value.

However, Schmidt et al. showed that the driving pressure was not associated with hospital mortality in non-ARDS patients (include HF patients) using the MIMIC-II database for analyses [26]. In Schmidt's research, only 9.16% (57/622) of patients were diagnosed with cardiac-related diseases (myocardial infarction, acute coronary syndrome, and congestive HF). Therefore, their results do not represent the association between driving pressure and mortality in patients with HF. Moreover, they excluded patients who were transferred from other hospitals to eliminate patients ventilated before ICU admission [26], which may have changed the result. In sensitivity analysis, we also excluded 171 patients who were ventilated before ICU admission and the result remained stable. Besides, the MIMIC-III database is an extension of the MIMIC-II database; it incorporates the data contained in the old version (collected between 2001 and 2008) and augments it with newly collected data between 2008 and 2012. Our study included 632 patients with HF whose data were analyzed and adjusted for 20 potential confounding factors. The relationship between driving pressure and mortality in patients with HF was stable.

In our study, we followed Serpa Neto et al. 's method to include and exclude patients [25]. However, as there was no evidence to show that being ventilated through a tracheostomy cannula had any effect on driving pressure, we did not exclude these patients. In sensitivity analysis, no different effect was found after excluding the 28 patients who were ventilated through a tracheostomy cannula within 48 h of ICU admission.

There are several limitations to this study. First, conclusions can be generalized to the patients with HF who were ventilated for more than 48 h only. Second, as in all observational studies, there may have been some uncontrolled potential confounders. Besides, considering that the MIMIC-III database did not contain the diagnosis of right HF and the lack of patients diagnosed with cor pulmonale, it is impossible to exclude the right HF from our study. In the sensitivity analyses, we eliminated 47 patients with COPD who may have suffered undiagnosed cor pulmonale, and the association was still stable. Third, in terms of ventilation mode, a recent study conducted by Di Mussi et al. showed that, during pressure support ventilation (PSV), the increase in neuroventilatory drive could lead to the variations in respiratory drive and effort, which may have an impact on driving pressure [44]. In fact, we did not strictly limit to volume control ventilation, and some patients in our study received assisted ventilation mode, which imparted a certain bias to the results. However, there was no significant difference in ventilation patterns among groups at baseline and after adjusting for ventilation mode in multivariate analysis, and our results remained robust. In addition, due to the limitations of the database, there was no explanation about how the plateau pressure (Pplat) was measured and recorded. This should be carefully considered in the next step of our real-world research. Future studies will focus on random control trial researches to confirm the causal relationship between driving pressure and mortality.

## 5. Conclusion

There was a nonlinear relationship between driving pressure and mortality in patients with HF who were ventilated for more than 48 h, and this relationship was associated with increased in-hospital mortality when the driving pressure was more than 14.27 cmH_2_O.

## Figures and Tables

**Table 1:** Baseline characteristics of participants.

Covariate	Driving pressure			*P* value
	Low (5.00–12.85)	Mid (12.86–16.05)	High (16.10–35.68)	
	*n* = 217	*n* = 213	*n* = 202	
*Baseline characteristics*				
Age (years)	71.83 ± 14.55	72.20 ± 12.96	70.03 ± 12.76	0.216
Sex (male)	121 (55.76%)	110 (51.64%)	92 (45.54%)	0.11
BMI (kg/m^2^)	27.9 ± 8.0	29.7 ± 7.9	32.5 ± 9.7	<0.001
PBW (kg)	62.9 ± 11.2	62.2 ± 10.5	60.9 ± 11.1	0.163

*Disease severity score*				
SAPS	21.73 ± 4.27	22.43 ± 4.31	22.95 ± 4.58	0.017
SOFA	6.27 ± 3.27	6.48 ± 3.24	6.79 ± 3.14	0.18
Elixhauser index	11.00 (6.00–15.00)	8.00 (3.00–13.00)	10.00 (6.00–15.00)	0.01

*Vital signs*				
Heart rate (bpm)	85.03 ± 16.42	84.97 ± 16.40	86.85 ± 16.71	0.421
Respiratory rate (bpm)	18.74 ± 3.90	19.03 ± 3.80	18.97 ± 4.30	0.727
MAP (mmHg)	77.59 ± 11.35	75.47 ± 10.23	76.92 ± 9.56	0.099
CVP (cmH_2_O)	15.00 (12.00–19.50)	17.00 (14.00–23.00)	20.00 (16.00–26.00)	<0.001

*Laboratory examination*				
WBC (×10^9^/L)	14.99 ± 7.23	15.58 ± 7.31	16.31 ± 16.92	0.856
Hemoglobin (g/L)	12.26 ± 2.06	11.83 ± 2.05	11.68 ± 1.90	0.01
NT-proBNP (pg/ml)	5016.00 (1341.50–15748.25)	3753.00 (1344.50–6903.50)	4293.50 (2142.25–17458.50)	0.425
Creatinine (mg/ml)	1.53 ± 1.82	1.41 ± 1.24	1.46 ± 1.07	0.626
PH	7.29 ± 0.11	7.29 ± 0.11	7.28 ± 0.10	0.802
PO_2_ (mmHg)	79.00 (63.00–115.00)	80.00 (66.00–104.00)	76.50 (61.75–99.00)	0.239
PCO_2_ (mmHg)	53.41 ± 20.43	54.11 ± 20.48	57.65 ± 20.33	0.081
PF ratio	166.67 (108.54–245.83)	153.25 (106.87–240.00)	156.33 (106.79–218.78)	0.67

*Parameters of mechanical ventilation*				
PEEP (cmH_2_O)	7.36 ± 3.16	7.22 ± 3.21	6.64 ± 2.78	0.04
VT/PBW	8.29 ± 1.86	8.77 ± 1.66	9.22 ± 2.35	<0.001
CMV	77 (36.5%)	75 (35.7%)	74 (35.1%)	0.996

*Outcome*				
In-hospital mortality	59 (27.19%)	45 (21.13%)	77 (38.12%)	<0.001

BMI: body mass index, PBW: predicted body weight, SOFA score: sequential organ failure assessment score, SAPS score: simplified acute physiology score, MAP: mean arterial pressure, CVP: central venous pressure, PEEP: positive end-expiratory pressure, bpm: beats per minute, WBC: white blood cell, NT-proBNP: N-terminal pro-B-type natriuretic peptide, PaO_2_: partial pressure of oxygen in arterial blood, PaCO_2_: partial pressure of carbon dioxide in arterial blood, PF ratio: PaO_2_/FiO_2_ ratio, VT/PBW: tidal volume/predicted body weight, and CMV: control mechanical ventilation.

**Table 2:** Multivariable logistic regression analyses of driving pressure and in-hospital mortality.

Variable	Crude model, *n* = 632	*P* value	Minimally adjusted model, *n* = 632	*P* value	Fully adjusted model, *n* = 603	*P* value
Driving pressure (cmH_2_O)	1.07 (1.02, 1.11)	0.0022	1.09 (1.04, 1.14)	0.0002	1.12 (1.06, 1.18)	<0.001

Driving pressure tertials						
Low	Reference		Reference		Reference	
Mid	0.72 (0.46, 1.12)	0.1430	0.71 (0.45, 1.12)	0.1376	0.74 (0.44, 1.23)	0.242
High	1.65 (1.09, 2.49)	0.0174	1.89 (1.23, 2.90)	0.0039	2.26 (1.36, 3.77)	0.002
*P* for trend	0.009	0.0019	0.002			

Crude model: no other covariates were adjusted. Minimally adjusted model: we adjusted age and sex. Fully adjusted model: we adjusted age, sex, BMI, heart rate, MAP, WBC, hemoglobin, creatinine, CVP, NT-proBNP, PEEP, PH, PaO_2_, PaCO_2_, PF ratio, ventilation mode (CMV), VT/PBW, Elixhauser score, SOFA score, and SAPS score.

**Table 3:** Threshold effect analysis of driving pressure on in-hospital mortality.

Threshold of driving pressure	OR	95% CI	*P* value
<14.27 cmH_2_O	0.89	0.75–1.05	0.176
≥14.27 cmH_2_O	1.17	1.07–1.30	<0.001

Adjustment factors included age, sex, BMI, heart rate, MAP, WBC, hemoglobin, creatinine, CVP, NT-proBNP, PEEP, PH, PaO_2_, PaCO_2_, PF ratio, ventilation mode (CMV), VT/PBW, Elixhauser score, SOFA score, and SAPS score.

## Data Availability

All data in the article can be obtained from the MIMIC-III database (https://mimic.physionet.org/).

## Abbreviations

ARDS: Acute respiratory distress syndrome
bpm: Beats per minute
BMI: Body mass index
CVP: Central venous pressure
CMV: Control mechanical ventilation
HF: Heart failure
ICU: Intensive care unit
MAP: Mean arterial pressure
MIMIC-III: Medical Information Mart for Intensive Care-III
NT-proBNP: N-Terminal pro-B-type natriuretic peptide
PaCO_2_: Partial pressure of carbon dioxide in arterial blood
PaO_2_: Partial pressure of oxygen in arterial blood
PBW: Predicted body weight
PF ratio: PaO_2_/FiO_2_ ratio
PEEP: Positive end-expiratory pressure
SAPS score: Simplified acute physiology score
SOFA score: Sequential organ failure assessment score
VT/PBW: Tidal volume/predicted body weight.

## Supplementary Materials

Supplementary Materials Supplementary Materials: additional DOC file includes supplementary tables showing the results of the sensitivity analyses.
