# Supplementary material for: Relationship between Driving Pressure and Mortality in Ventilated Patients with Heart Failure: A Cohort Study
Source: Can Respir J. 2021 Nov 29;2021:5574963. doi: 10.1155/2021/5574963 (PMC8648448; doi:10.1155/2021/5574963)
Supplement: Supplementary Materials — additional DOC file includes supplementary tables showing the results of the sensitivity analyses. [file 5574963.f1.doc]

**Supplementary Table 1. The results of univariate and multivariate analysis**

| **Exposure** | **Univariate**  (OR,95%CI,*P*) | **Multivariate**  (OR,95%CI,*P*) |
| --- | --- | --- |
| Age (years) | 1.04 (1.03, 1.06) <0.0001 | 1.04 (1.02, 1.06) <0.0001 |
| Gender (male) | 0.90 (0.64, 1.27) 0.5374 | 0.90 (0.60, 1.35) 0.6185 |
| SAPS | 1.09 (1.05, 1.13) <0.0001 | 1.05 (0.99, 1.11) 0.0846 |
| SOFA | 1.05 (0.99, 1.10) 0.0903 | 1.08 (0.99, 1.17) 0.0853 |
| Elixhauser index | 1.03 (1.00, 1.05) 0.0551 | 1.03 (1.00, 1.06) 0.0968 |
| Heart rate (bpm) | 1.00 (0.99, 1.01) 0.4099 | 1.00 (0.99, 1.01) 0.6238 |
| Respiratory rate (bpm) | 1.02 (0.98, 1.07) 0.2619 | 1.03 (0.98, 1.08) 0.2491 |
| Blood pressure (mmHg) | 0.99 (0.98, 1.01) 0.3468 | 1.01 (0.99, 1.03) 0.4288 |
| CVP(Yes) | 1.00 (0.70, 1.45) 0.9794 | 0.97 (0.64, 1.47) 0.8956 |
| PEEP (cmH2O) | 0.97 (0.92, 1.03) 0.3626 | 1.02 (0.94, 1.09) 0.6588 |
| Driving pressure | 1.07 (1.02, 1.11) 0.0022 | 1.10 (1.05, 1.16) <0.0001 |
| WBC | 1.01 (0.99, 1.02) 0.2555 | 1.02 (1.00, 1.05) 0.0951 |
| Hemoglobin (g/L) | 0.97 (0.89, 1.05) 0.4535 | 1.03 (0.93, 1.15) 0.5466 |
| PH | 0.85 (0.17, 4.17) 0.8397 | 4.53 (0.41, 49.76) 0.2164 |
| PO2 (mmHg) | 1.00 (1.00, 1.00) 0.1910 | 1.00 (0.99, 1.00) 0.8384 |
| PCO2 (mmHg) | 1.00 (0.99, 1.01) 0.7278 | 1.01 (0.99, 1.02) 0.3059 |
| PF ratio | 1.00 (1.00, 1.00) 0.5182 | 1.00 (1.00, 1.00) 0.3298 |
| NT-proBNP(Yes) | 0.82 (0.52, 1.29) 0.3921 | 0.78 (0.47, 1.30) 0.3375 |
| Creatinine(mg/ml) | 0.98 (0.86, 1.11) 0.7512 | 0.91 (0.75, 1.10) 0.3302 |

**Supplementary Table 2.** **Multivariable logistic regression analyses of driving pressure and in-hospital mortality after exclude the patients who receiving ventilation through a tracheostomy cannula at any time during the first 48 hours of ventilation**

| **Exposure** | **Crude model**  **N=604** | **Minimally adjusted model N=604** | **Fully adjusted model** |
| --- | --- | --- | --- |
|  |  |  | **N=577** |
| **Driving pressure (cmH2O)** | 1.07 (1.02, 1.11) 0.0026 | 1.09 (1.04, 1.14) 0.0002 | 1.11 (1.05, 1.16) <0.0001 |
| **Driving pressure tertiles** |  |  |  |
| **Low** | Reference | Reference | Reference |
| **Mid** | 0.72 (0.45, 1.13) 0.1495 | 0.72 (0.45, 1.15) 0.1669 | 0.65 (0.39, 1.08) 0.0931 |
| **High** | 1.65 (1.08, 2.52) 0.0198 | 1.93 (1.24, 3.01) 0.0035 | 2.08 (1.27, 3.40) 0.0036 |

**Supplementary Table 3. Multivariable logistic regression analyses of driving pressure and in-hospital mortality after exclude patients ventilated before ICU admission**

| **Exposure** | **Crude model** | **Minimally adjusted model N=461** | **Fully adjusted model N=434** |
| --- | --- | --- | --- |
|  | **N=461** |  |  |
| **Driving pressure (cmH_2_O)** | 1.07 (1.01, 1.12) 0.0112 | 1.09 (1.03, 1.15) 0.0016 | 1.10 (1.03, 1.17) 0.0020 |
| **Driving pressure tertiles** |  |  |  |
| **Low** | Reference | Reference | Reference |
| **Mid** | 0.73 (0.43, 1.24) 0.2418 | 0.70 (0.41, 1.20) 0.1987 | 0.68 (0.37, 1.23) 0.2038 |
| **High** | 1.53 (0.94, 2.50) 0.0871 | 1.80 (1.08, 2.99) 0.0249 | 1.84 (1.02, 3.29) 0.0414 |

**Supplementary Table 4. Multivariable logistic regression analyses of driving pressure and in-hospital mortality after exclude the COPD patients**

| **Exposure** | **Crude model** | **Minimally adjusted model N=585** | **Fully adjusted model N=557** |
| --- | --- | --- | --- |
|  | **N=585** |  |  |
| **Driving pressure(cmH2O)** | 1.06 (1.02, 1.11) 0.0072 | 1.08 (1.03, 1.13) 0.0007 | 1.10 (1.05, 1.16) 0.0002 |
| **Driving pressure tertiles** |  |  |  |
| **Low** | Reference | Reference | Reference |
| **Mid** | 0.70 (0.44, 1.10) 0.1231 | 0.69 (0.43, 1.11) 0.1249 | 0.68 (0.41, 1.14) 0.1402 |
| **High** | 1.62 (1.05, 2.48) 0.0282 | 1.87 (1.20, 2.93) 0.0060 | 2.16 (1.30, 3.58) 0.0029 |
